# Food and beverage purchases at formal and informal outlets in Mexico

**DOI:** 10.1017/S1368980022002324

**Published:** 2023-05

**Authors:** Irene Farah, Dalia Stern, Yenisei Ramírez, Nancy López-Olmedo, Carolina Pérez-Ferrer, Brent A Langellier, M Arantxa Colchero, Tonatiuh Barrientos-Gutierrez

**Affiliations:** 1 Department of City and Regional Planning, University of California at Berkeley, Berkeley, CA, USA; 2 CONACyT–Center for Research on Population Health, National Institute of Public Health, Cuernavaca, Morelos, Mexico; 3 Center for Research on Population Health, National Institute of Public Health, Cuernavaca, Morelos, Mexico; 4 CONACyT–Center for Research on Nutrition and Health, National Institute of Public Health, Cuernavaca, Morelos, Mexico; 5 Dornsife School of Public Health, Drexel University, Philadelphia, PA, USA; 6 Center for Research on Health Systems, National Institute of Public Health, Cuernavaca, Morelos, Mexico

**Keywords:** Mexico, Food purchases, Informality, Outlet-type, Income, Urbanicity

## Abstract

**Objective:**

To examine food and beverage purchasing patterns across formal and informal outlets among Mexican households’ and explore differences by urbanicity and income.

**Design:**

Cross-sectional study of a nationally representative sample of households. We calculated the proportion of total food and beverage expenditure in each household by food outlet type overall and by urbanicity and income. We defined informal outlets as those which are not registered or regulated by tax and fiscal laws. Since some of the outlets within community food environments do not fall in clear categories, we defined a continuum from formal to informal outlets, adding mixed outlets as a category.

**Setting:**

Mexico.

**Participants:**

Mexican households (*n* 74 203) from the 2018 National Income and Expenditure Survey.

**Results:**

Of the total food and beverage purchases, outlets within the formal food sector (i.e. supermarkets and convenience stores) accounted for 15 % of the purchases, 13 % of purchases occurred in outlets within the informal food sector (i.e. street markets, street vendors and acquaintances) and 70 % in fiscally mixed outlets (i.e. small neighbourhood stores, specialty stores and public markets). Across levels of urbanicity and income, most food and beverage purchases occurred in mixed outlets. Also, purchases in informal and mixed outlets decreased as levels of urbanicity and income increased. In contrast to informal outlets, purchases in formal outlets were most likely from richer households and living in larger sized cities.

**Conclusions:**

Understanding where Mexican households shop for food is relevant to create tailored interventions according to food outlet type, accounting for regulatory and governance structures.

A promising approach to improving population-level dietary patterns and associated health outcomes is to intervene in the community food environment, that is, the environment in which food purchasing decisions are made^(1)^. Public policies aimed at regulating food outlets could contribute to increasing healthy food access and decrease the burden of obesity and obesity-related non-communicable diseases^(2)^. Several studies have analysed the link between the food environment, diet quality, obesity and non-communicable diseases^(3,4)^. Yet, community food environment research has predominantly been undertaken in high-income countries, where most food is sold in the formal sector, such as supermarkets, grocery stores and convenience stores. While low- and middle-income countries have experienced an increasing penetration of formal food outlets^(5,6)^, the informal food environment remains a critical component of the community food environment^(7–9)^; however, it is rarely described or studied.

Informal food vendors are highly heterogeneous, ranging from highly mobile solitary vendors of a single food to well-organised itinerant street markets^(10)^. Community food environment research in low- and middle-income countries, such as Latin America, has been scarce and has focused on formal environments due to lack of data availability^(10–15)^. While several dietary intake and food purchasing studies show that informal vendors make a significant contribution to energy and protein intake in low- and middle-income countries^(8,16)^, the majority of research on the informal food sector focuses on food safety and contamination^(17)^. Most studies recognise the lack of information on informal food markets as a major limitation^(10,18,19)^. Studies showing that informal food outlets are an important source of food provision are predominantly focused on disadvantaged populations in big cities^(9,16,18,20)^ and are limited in geographical scope^(17)^. Most of these studies have focused on specific cities and have not examined empirically for the entire Mexican population how these food consumption patterns vary across households’ socio-economic status and urbanicity. In particular, research on food consumption and urbanisation in low- and middle-income countries have found that differences across the rural–urban continuum behave similarly to socio-economic status, finding that urban areas consume more from formal outlets^(21,22)^; however, these studies have not included informal food and beverage outlets.

Beyond informal food outlets, recent work has also emphasised the importance of mixed outlets, understood as small, family-owned outlets that are widespread in low- and middle-income countries, in contrast to supermarkets or chain convenience stores^(5,18,23–26)^. While the national statistics bureaus attempt to quantify the percentage of the Gross Domestic Product that belongs to the informal sector^(27)^; these small, family-owned establishments are not defined within sectors, depicting the inaccuracy and complication of the binary category of informality. These studies recognise that compared with cities in the USA, there is a multiplicity of food and beverage outlets that are ubiquitous and of high cultural and economic importance. Nevertheless, these studies are either limited in geographic scope or have only focused on packaged food purchases, neglecting produce purchases, products sold in bulk and products sold in informal outlets.

Mexico, like many other Latin American countries, has implemented food pricing as well as food labelling policies to improve diet healthfulness at the population level^(28)^. However, no policies have been implemented to regulate the quality of food retailers^(29)^. Due to their regulatory nature, policies that affect formal food retailers are easier to implement than policies focused on the informal sector^(7)^. While the informal food sector seems to be an important source of food for the urban poor^(7,9,20)^, we do not know to what extent people in different urban and socio-economic strata purchase their foods in the formal, mixed and informal food sector. By consequence, the effectiveness of food retailer policies may vary across different social groups. For example, policies that can only be implemented in the formal sector may not have an impact on people that primarily shop in the informal sector. Thus, addressing this knowledge gap can lead to more effective, equitable food policy.

We used the National Income and Expenditure Survey, known as *Encuesta Nacional de Ingreso Gasto de los Hogares* (ENIGH) from 2018, a nationally representative sample of Mexican households. ENIGH is unique for studying food purchases across food and beverage outlets, including the informal and fiscally mixed food sector. In this descriptive analysis, we addressed two research questions: (1) in which food outlets do Mexican households shop for food and beverages? and (2) does shopping in different food outlets vary by household income and urbanicity?

## Methods

### Data sources

We used data from the 2018 ENIGH, conducted by the National Institute of Statistics and Geography of Mexico (INEGI). The ENIGH is a probabilistic survey with a two-stage stratified clustered sampling design, representative at the national and state levels, and urban and rural strata. It includes data on household expenditures in food and beverages and socio-demographic characteristics such as income and place of residence^(30)^. The 2018 ENIGH was collected between August and November. Detailed information about data collection is available elsewhere^(31)^.

Data collectors visited each household daily for seven consecutive days to collect information on food and beverage purchases^(30)^. Household purchases were reported by the household member responsible for purchasing food and beverages and complemented by individual members using a food diary. The food and beverage diary compiles data on the name of the food or beverage, quantity purchased (litres and kilograms), expenditures (Mexican pesos) and the type of food outlet where the purchase was made. ENIGH also collects information on household expenditures on food consumed away from home, such as restaurants or food services^(30)^.

ENIGH 2018 included purchases for 74 647 households. We excluded households that did not report any food and beverage monetary purchases (*n* 438). We also excluded households that only reported purchases of animal feed, cigars, cigarettes and tobacco (*n* 6). The final sample size was 74 203 households.

### Type of food and beverage outlets

ENIGH categorises Mexico’s wide variety of food and beverage outlets into the following eighteen categories^(30)^: street vendors, street markets (*tianguis*), acquaintances (people that sell foods to neighbours, friends, family or workplaces), public markets, small neighbourhood stores (*abarrotes*), specialty stores, low-budget restaurants (*fondas, cocinas económicas, loncherías*), restaurants, cafeterias, bars, convenience stores, supermarkets, wholesale stores, department stores, government-owned stores, private companies where workers eat, community kitchens and government social programmes, purchases made outside the country and food sold through the Internet (Table 1).

**Table 1:** Characteristics of food and beverage outlets in Mexico

Food and beverage outlets	Characteristics	Fiscal regime*
Street vendors	Improvised stalls on public space, mobile vendors or vendors selling from home to home.	Informal
Street markets *(i.e. tianguis)*	Open-air street market with vendors selling on a specific day of the week (not a fixed establishment).	
Acquaintances	People that sell foods to neighbours, friends, family or workplaces.	
Public markets	Enclosed public space with formal and informal vendors selling daily in fixed establishments.	Mixed
Small neighbourhood stores (i.e. *abarrotes*)	Small neighbourhood retail stores that sell multiple products. Similar to *bodegas* in the USA. Their fiscal regime could be formal or informal.	
Specialty stores	Establishments that sell only one product. Most commonly they are outlets selling tortillas, fruits and vegetables, poultry, fish/seafood, dairy or butchers.	
Low-budget restaurants (i.e. *fonda, cocina económica, lonchería and taquería*)	Small, public establishments that sell prepared foods and are consumed *in situ*, offer low-budget, affordable meals, do not sell alcoholic beverages, usually do not accept credit cards and the selection of foods is restricted to specific meals.	
Restaurants	Public establishments that sell prepared foods and are consumed *in situ*, they can offer alcoholic beverages, accept credit cards and offer a menu.	Formal
Cafes	Public establishments that offer mostly coffee and pastries.	
Bars	Public establishments (including *cantinas* and *pulquerías*) that offer alcoholic beverages.	
Convenience stores	Small retail chain establishment that sells everyday products (e.g. *7-eleven*, *Oxxo*).	
Supermarkets	Large chain retail establishments divided by specialised departments (e.g. *Walmart*, *Comercial Mexicana*).	
Wholesalers	Large retail that requires membership (e.g. *Costco, Sam’s Club*).	
Department stores	Large establishments with specialised departments that exclude fresh and perishable foods (e.g. *Sanborns*, *Palacio de Hierro*).	
Private	Private companies that offer food to their workers.	
Government	Establishments that sell foods and milk at a subsidised price with the goal to alleviate food insecurity (e.g. *Diconsa*, *Liconsa*).	
International	Purchases made outside the country (usually bought on border towns).	
Internet	Purchases made through the Internet.	

* Informal outlets are those that either production or employment is unregistered or without social protection. Formal outlets are registered establishments in terms of production and employment, with workers having social protection. Mixed outlets could fall in either categories since we cannot assure if they are registered or not.

**Table 2:** Socio-demographic characteristics of Mexican households, ENIGH 2018 (*n* 74 203)

Income		*n*	%
National	Q1	6 918 056	20·0
	Q2	6 918 056	20·0
	Q3	6 918 056	20·0
	Q4	6 918 056	20·0
	Q5	6 918 056	20·0
Urbanicity*			
Rural		7 968 951	23·0
Small		4 851 874	14·0
Medium		5 087 952	14·7
Metropolitan		16 681 503	48·2
Urbanicity and income			
Rural	Q1	1 593 790	4·6
	Q2	1 593 790	4·6
	Q3	1 593 790	4·6
	Q4	1 593 790	4·6
	Q5	1 593 791	4·6
Small	Q1	970 375	2·8
	Q2	970 375	2·8
	Q3	970 375	2·8
	Q4	970 375	2·8
	Q5	970 374	2·8
Medium	Q1	1 017 590	2·9
	Q2	1 017 590	2·9
	Q3	1 017 590	2·9
	Q4	1 017 590	2·9
	Q5	1 017 592	2·9
Metropolitan	Q1	3 336 300	9·6
	Q2	3 336 300	9·6
	Q3	3 336 300	9·6
	Q4	3 336 300	9·6
	Q5	3 336 303	9·6

* Rural < 2500 inhabitants; small cities 2500–14 999 inhabitants; medium-sized cities 15 000–99 999 inhabitants and metropolitan cities ≥100 000 inhabitants.

Values represent column % for income and urbanicity.

The percentages by income are estimated from the total population of households (34 590 280).

Survey commands were used to account for survey design and weighting to generate nationally representative results.

Since some of these categories accounted for a small percentage of the total purchases, we aggregated food outlets into eleven mutually exclusive categories with similar features: (1) street vendors; (2) street markets; (3) acquaintances; (4) public markets; (5) small neighbourhood stores; (6) specialty stores; (7) low-budget restaurants; (8) restaurants, cafes and bars; (9) convenience stores; (10) supermarkets and (11) others (i.e. wholesalers, department stores, private and government outlets, and international and internet purchases).

### Formal, informal and mixed food and beverage outlets

The ENIGH questionnaire captures purchases from the formal food sector and the informal food sector although they do not classify the outlets as such. Since the only information we have from ENIGH is the type of store, our classification into informal and formal outlets was limited. We classified supermarkets, convenience stores, and restaurants, cafes and bars as formal outlets, and street vendors, street markets, and acquaintances as informal outlets. This decision was informed by the International Labour Office’s (ILO) broad definition of informality as the production and employment carried out in unregistered establishments and without social protection^(32)^. Broadly, most street vendors, street markets and acquaintances engage in operations that are not registered or regulated by fiscal and labour laws^(32)^. Over the past 17 years, informal labour has accounted for 21·9 %–24·4 % of the Gross Domestic Product ^(27)^ in Mexico, although this includes workers within the formal sector as well. Some public markets, small neighbourhood stores, specialty stores and low-budget restaurants might also be fiscally unregulated, but we chose not to define these categories as informal because many businesses in these categories are regulated. While there are establishments that pay some taxes or have employees that have social security, it is more likely that our definition is conservative since there have been recent efforts to formalise small neighbourhood stores and specialty stores providing incentives for outlets that do not pay taxes and where employees do not have social security^(33)^. While we are aware that clear-cut definitions of informality are *ad hoc*, we decided to include this approach since there is little evidence of Mexicans’ food purchasing patterns in these outlets. Thus, we categorised public markets, small neighbourhood stores, specialty stores and low-budget restaurants as mixed food and beverage outlets since many of these outlets are small, family-owned businesses and could be either formal or informal outlets (Table 1)^(18,23–25)^.

### Urbanicity and income level

ENIGH classifies localities as rural if they have less than 2500 inhabitants, small cities if they have a population between 2500 and 14 999 people, medium-sized cities with 15 000 to 99 999 people, and metropolitan cities with more than 100 000 inhabitants. Quarterly household income was used as a proxy for socio-economic status. As shown in Table 2, income was classified into quintile groups, stratified from the poorest 20 % to the richest 20 %, accounting for the expansion weights of the survey^(34)^. Quintile groups were created for each urbanicity separately to be able to compare cities of the same size and account for socio-economic differences within each locality (see mean income for each quintile in Supplemental Table S1 and Table 2).

### Statistical analysis

To understand at what food outlets Mexican households shop, we calculated the proportion of total food expenditure by food outlet type. Households with no purchases in a given outlet were included in the analysis with a percentage contribution of zero. We also conducted this analysis across strata defined by income and urbanicity. Since household size might influence the proportion of food purchases by outlet where households shop for food, we estimated the percentage of food purchases by household size. We tested for statistically significant differences using Student’s *t*-test with the Bonferroni’s correction for multiple comparisons with a two-sided *P* value of 0·001 denoting statistical significance. We compared the values of each outlet against all other outlets at a national scale and within urbanicity and income levels. All analyses were conducted using the survey library^(35)^ in R^(36)^ to account for survey design and weights to generate nationally representative results. Results of standard errors are found in the supplementary section.

## Results

### Food and beverage outlet contribution to total food purchases

**Fig. 1:**
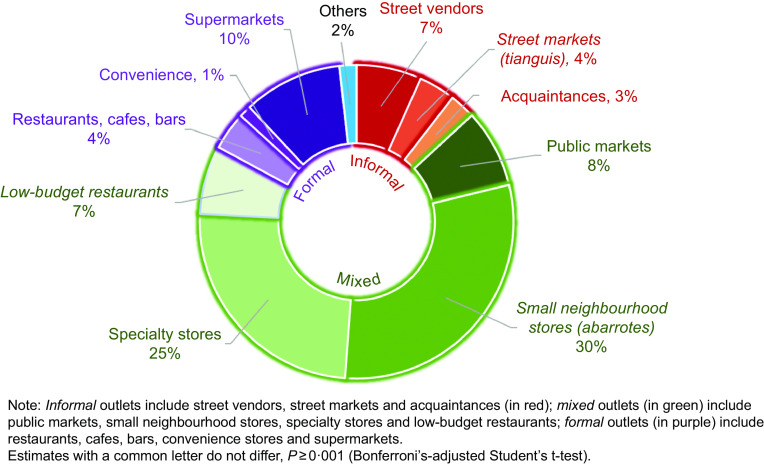
Households’ food and beverage purchases (% expenditure) by food outlet, ENIGH 2018

Figure 1 shows the contribution of household food and beverage purchases (proportional expenditure) by the type of outlet in 2018. The formal sector included supermarkets (accounted for 10 % of expenditure), restaurants, cafes and bars (4 %), and convenience stores (1 %). The informal food sector included household food purchases from street vendors (7 %), street markets (4 %) and acquaintances (3 %). The largest proportion of food and beverages was purchased at mixed outlets, accounting for 70 % of food and beverage household purchases (30 % in small neighbourhood stores, 25 % in specialty stores, 8 % in public markets and 7 % in low-budget restaurants).

### Food and beverage outlet contribution to total food purchases by urbanicity and income level

**Fig. 2:**
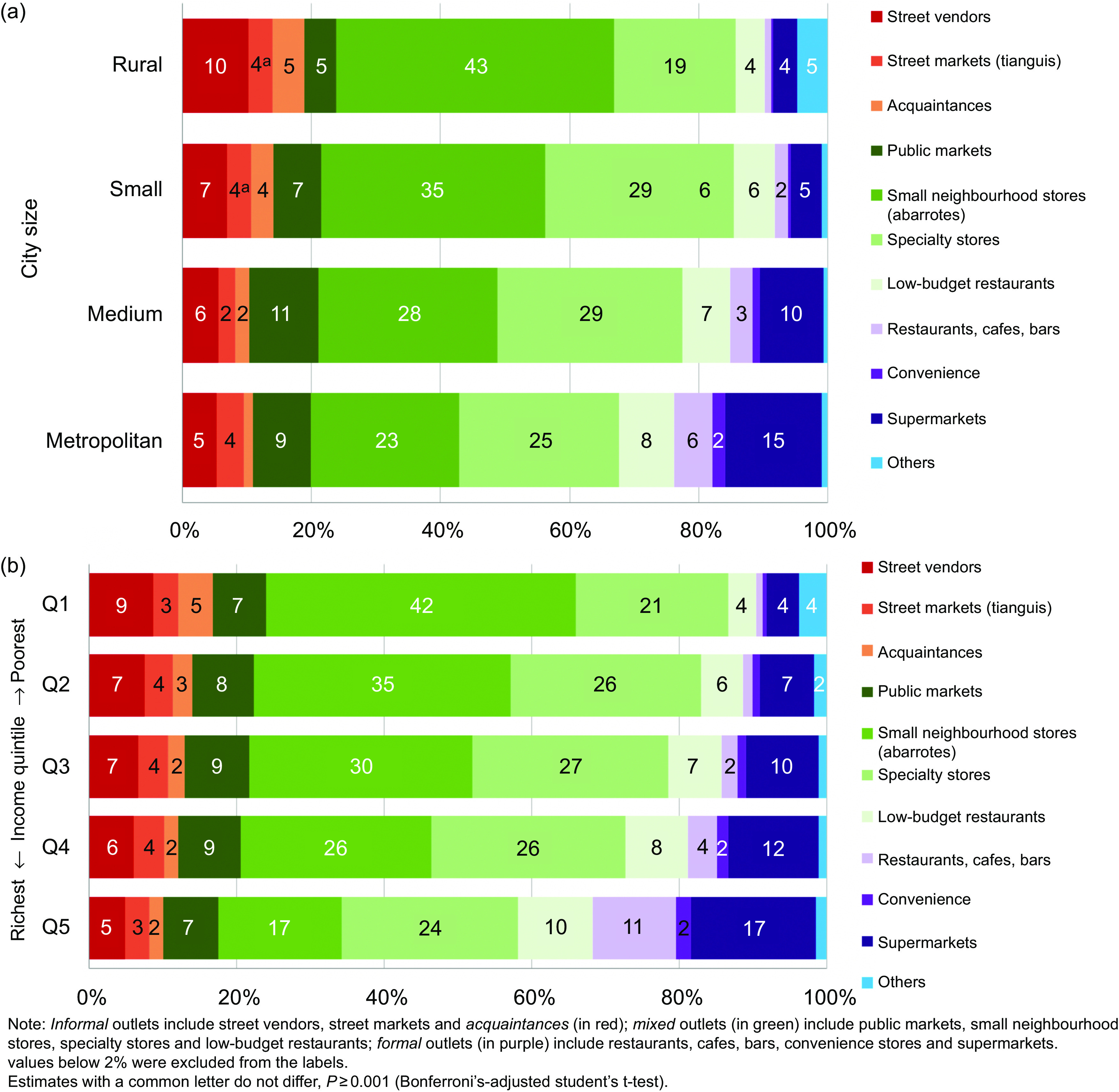
Households’ food and beverage purchases (% expenditure) by food outlet, ENIGH 2018. (a) urbanicity; (b) income level

Figure 2 shows the distribution of food and beverages purchases by food outlet, stratified by urbanicity (Fig. 2(a)) and income level (Fig. 2(b)). Figure 2(a) shows a higher purchase share of foods and beverages from street vendors, small neighbourhood stores, acquaintances and other outlets in smaller cities, on average. All comparisons were statistically significantly different except comparisons between street markets in rural and small cities. In rural areas, street vendors and small neighbourhood stores accounted for 10 % and 43 % of total food expenditure, respectively, compared with 5 % and 23 % in metropolitan areas. Acquaintances and other outlets contributed 5 % of money spent in rural areas compared with 1 % in metropolitan areas since these outlets comprise mostly government outlets and community kitchens. The opposite trend was observed for supermarkets, convenience stores, and restaurants, cafes and bars. In rural areas, 4 % of purchases occurred in supermarkets and approximately 1 % in convenience stores and restaurants, cafes and bars on average, compared with 15 %, 2 % and 6 % respectively, in metropolitan areas. Purchases in specialty stores, public markets and low-budget restaurants were more prevalent in larger cities, while purchases in street markets were similar across urbanicity.

The patterns were similar for income groups. As shown in Fig. 2(b), households with the lowest income made, on average, 42 % of their food and beverage purchases in small neighbourhood stores, compared with 17 % in the highest income quintile. In contrast, households from the lowest quintile bought, on average, 4 % from supermarkets, compared with 17 % of households in the highest income quintile. Furthermore, while lowest income households bought on average 1 % of their food and beverage purchases in convenience stores, high-income households purchased 9 % of their foods from street vendors. In comparison, the highest quintile group bought on average 2 % of their food purchases in convenience stores and 5 % from street vendors. Food purchases in specialty stores, street markets and public markets behaved similarly across income groups. Purchases in restaurants, cafes and bars were higher for the highest quintile group, representing on average 11 % of their food and beverage purchases, compared with 1 % for the poorest income group. Similarly, low-budget restaurants contributed in 10 % in the highest income group, compared with 4 % in the lowest socio-economic group. We made a distinction between restaurants and low-budget restaurants because restaurants are more expensive than low-budget restaurants that offer affordable meals for the working class. This does not mean, however, that households belonging to the lowest income levels can afford or attend these establishments, since eating out is usually more expensive.

We also estimated the percentage of food purchases by household size to assess how household size might influence the proportion of food purchases by outlet where households shop for food (see online Supplemental Fig. S1). Since the results do not show dramatic differences by household size when looking at the relative contribution of each food outlet to total purchases by income and urbanicity, we did not include household size in the analysis.

**Fig. 3:**
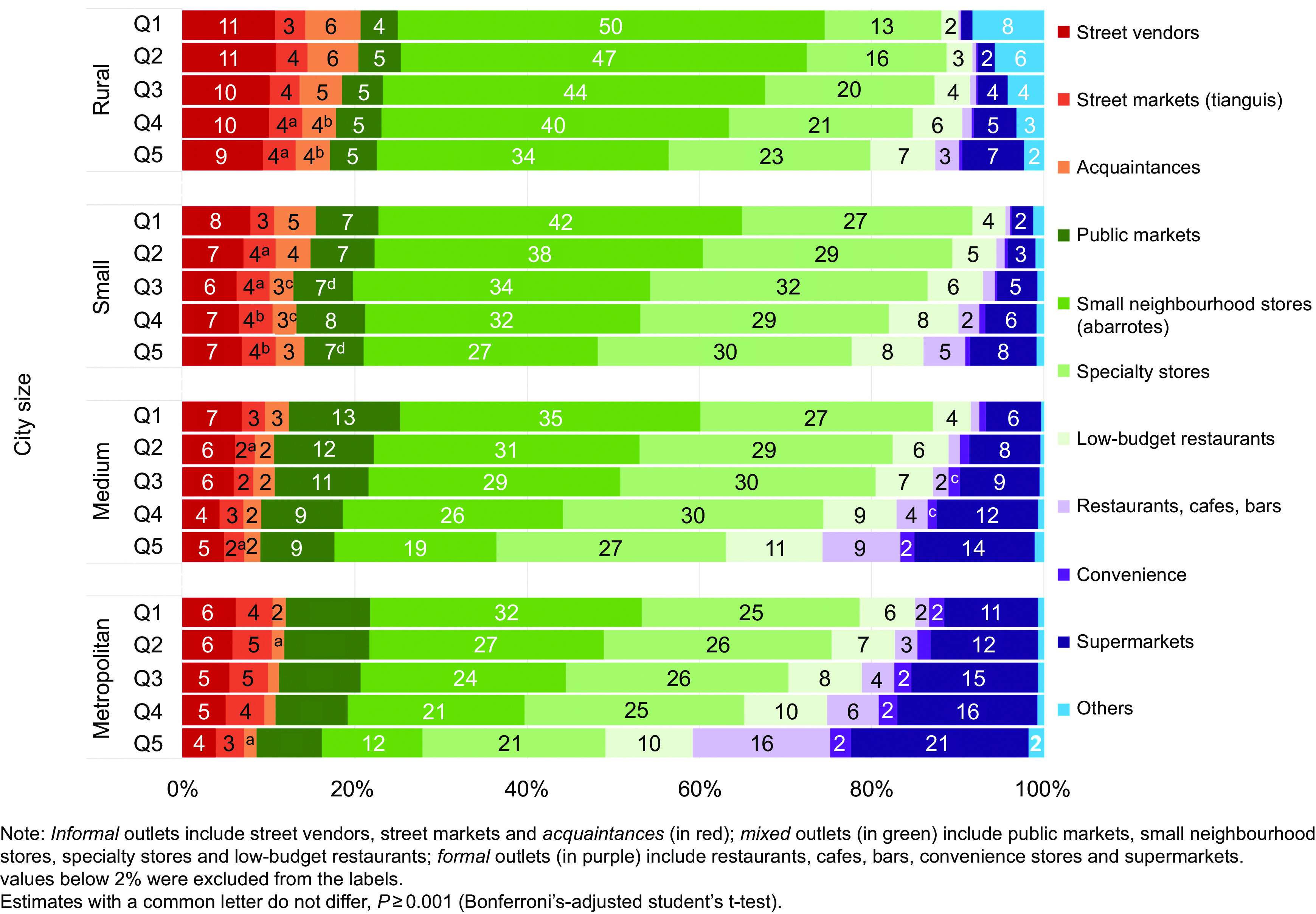
Households’ food and beverage purchases (% expenditure) by food outlet, urbanicity and income, ENIGH 2018

Figure 3 combines socio-economic group and urbanicity data, showing that households in rural areas and within the lowest income group had the largest percentage (20 %) of purchases in informal food outlets (i.e. street vendors, street markets and acquaintances) in contrast to households in metropolitan areas and within the highest income group (8 %). Figure 3 also shows that the average percentage of purchases for small neighbourhood stores decreases as the household income increases similarly across all urbanicities. Supermarkets show an opposite pattern, having the lowest percentage for the poorest households in rural areas (1 %) and the highest percentage for the richest households in metropolitan areas (21 %). Similarly, restaurants, cafes and bars have a negligible contribution in rural and lower income households (<1 %), compared with higher income households living in metropolitan areas (16 %). Specialty stores are commonly available and relatively high across all income groups and urbanicity level. Additionally, public markets do not show a clear pattern across urbanicity except for a slightly greater prevalence in lower income households within medium (13 %) and metropolitan cities (10 %). Furthermore, convenience stores show a greater contribution (7 %) for the highest income group in the metropolitan areas, while its contribution is negligible in the lowest income groups of the smaller city and rural categories.

## Discussion

Using data from a nationally representative sample of Mexican households, we found that mixed outlets (i.e. public markets, small neighbourhood stores, specialty stores and low-budget restaurants) are a very important source of food purchases in Mexico, accounting for 70 % of households food and beverage purchases. These outlets also make up the greatest fraction of reported food and beverage expenditures for households in small cities and within the lowest income group. This pattern also holds when we analyse purchases within each urbanicity, showing that the largest percentage of food purchases in mixed outlets is among the lowest income households. The informal food sector accounts for 13 % of food and beverage purchases. Interestingly, our results do not show any clear pattern across income groups within each urbanicity, despite the belief that poorer households buy greater percentages of food from the informal food sector^(16,18,20)^. Within the formal sector, restaurants, cafes and bars, supermarkets, and convenience stores account for 15 % of food and beverage purchases, showing clear patterns of increasing proportions as the urbanicity increases and as households become richer.

Our findings are consistent with prior studies from Latin America that show that the majority of food and beverage purchases occur in mixed food and beverage outlets^(11,13,23–26,37,38)^. Previous works from Pedraza and her co-authors^(23–25)^ have also studied the variation of purchases across store-type, including mixed outlets (which the authors identify as ‘traditional’ stores) in their analyses and illustrating the multiplicity of outlets where Mexicans buy in. However, it is likely that these studies underestimated the proportion of purchases from mixed outlets since their data solely focused on packaged foods, excluding products like fresh produce, tortillas, meat or poultry, and ignoring the informal food sector. Moreover, they only studied areas with more than 50 000 inhabitants, overlooking how purchases vary across urbanicity. Other studies have also argued that the surge of formal retailers like supermarkets and convenience stores and the decline of informal outlets in low and middle-income countries is due to marketing by international and local supermarket chains and a notable increase of foreign direct investment^(5)^. However, some studies have been critical of the ‘supermarket revolution’ thesis and its negative impact on the informal food sector^(26,38–40)^.

Few studies have quantified the role of the informal food sector in contrast to supermarkets across countries, apart from the data collection effort from the African Food Security Urban Network and the Healthy Cities Partnership. Jonathan Crush and Bruce Frayne^(41)^ provide descriptive findings across South African cities, illustrating that around 70 % of households in their survey sourced their food from informal outlets. Their study also showed different product purchasing patterns such as bulk buying in supermarkets, while basic foodstuffs are mostly purchased in informal outlets. The Hungry Cities Partnership has studied the sources of food purchases and patronage in Bangalore (India), Cape Town (South Africa), Kingston (Jamaica), Maputo (Mozambique), Mexico City (Mexico), Nanjing (China) and Nairobi (Kenya). Using this data, these studies^(15,26)^ argue that formal and informal outlets coexist across cities, showing that high levels of supermarket patronage do not rule out high levels of patronage of informal food outlets. They also show that street vendors in Mexico City have the lowest percentage of patronage compared with the other cities. However, their definition of formal and informality differs from the one defined in our study, and they do not account for the quantity bought in each outlet. Other studies have also used these data to show the importance of supermarkets across cities in Africa^(14,42)^ and the prevalence of certain food products across different food outlets, like wet markets selling mostly fresh produce while supermarkets rely heavily on processed foods purchasing in China^(43)^. Likewise, some studies in the USA have shown the impacts that street vendors and farmers markets have on healthy food availability in lower income neighbourhoods^(44,45)^. Most importantly, the variation that the informal and formal food sectors play within and between cities in these studies highlights the importance of context-specific analyses to have a better understanding of food and beverage purchasing patterns.

While findings from Pérez-Ferrer and her co-authors^(38)^ also depict a large increase in the number of supermarkets and convenience stores from 2010 to 2016 in Mexico, they do not find a decline in small neighbourhood stores or specialty stores, exposing the resilience of the mixed food sector and the expansion of multiple food and beverage outlets. Similarly, our results show that despite the ‘modernisation’ of the food environment, the prevalence of mixed food and beverage purchases holds even for households in the highest income group and in the largest cities (43 %). Our results also portray gradients across income and urbanicity strata in formal and informal outlets showing that households in the poorest stratum in rural areas purchased more in informal outlets (20 %) and less in formal outlets (1 %) than the richest households in metropolitan areas (8 % in informal and 39 % in formal). However, a longitudinal analysis is needed to assess how fast purchases have been changing across the formal, mixed and informal sectors in the past 20 years. While we did not examine purchasing patterns longitudinally, this is the first study analysing food and beverage purchases which includes the informal food sector at a national scale. This finding is important since informal food outlets have been excluded from governmental censuses with little information gathered from these outlets.

In order to design programmes and public policies to improve the community food environment, we first need to understand where people purchase their food. Until now, most potential interventions are thought within the formal food sector; however, as our results show, people buying in these outlets are most likely from richer households and living in larger cities. The proportion of households in the highest income level and most urban level accounts for 9·6 % of the overall population. By disaggregating households by income and urbanicity, we show that households buying the largest proportion of foods at supermarkets account for a small percentage of the population. Thus, public policies neglecting the mixed and informal food sector are impacting a small proportion of the population and potentially promoting greater health disparities, targeting more advantaged populations who already have a lower burden of obesity and associated chronic diseases^(46)^. These results also reveal that in order to create equitable interventions, we cannot have one-size-fits-all policies across informal, mixed, formal outlets. As an example of such policies, the Tax Administration Service implemented the programme *Crezcamos Juntos* (Let’s Grow Together) to incentivise formalisation among small neighbourhood stores and specialised stores. The programme offered a year free of taxes and discounts within the first 10 years of registration. Registering these outlets within the taxation and social security systems is a first step to recognise the importance of these outlets within community food environments so policymakers can start thinking about regulation of quality of food within these outlets^(47)^. Policies intervening in the food environment must consider the cost, complexity of enforcement and existing governance structures across different food outlets. Our results provide a nuanced view of Mexico’s food environment, providing policymakers insights to create innovative policies accounting for outlets’ regulatory differences and conditions, since intervening an informal vendor is just as important as regulating a supermarket in terms of food and beverage purchases. Moreover, future studies should analyse which types of food and beverages are being purchased in each outlet to have more impactful policies.

Despite the richness of data provided by ENIGH, our analysis is not without limitations. First, data on food and beverage purchases are commonly underestimated^(48)^. It is likely that data on food and beverage purchases are underestimated differently across food outlets, underestimating household purchases in smaller outlets more than in larger outlets. Second, it is also important to acknowledge that purchases are not equivalent to consumption. Not all foods purchased at a store are consumed, and we were not able to account for food waste. In addition, if purchases vary across the year, the data may have seasonality issues since it only captures purchases from August to November.

However, the nutritional profile of purchases is correlated with diet quality as measured by 7 d, 24-h recalls and therefore a good representation of an entire week overall intake^(49,50)^. Third, we created an *ad hoc* categorisation of informal, mixed and formal outlets. Without an international standard definition of informality^(32)^, the categorisation of stores as formal, informal and mixed becomes complex and reflects the limitations of quantifying informality. The main challenge was that ENIGH does not provide the name of the establishments where purchases were made. The only information we had is the type of store, limiting our ability to determine whether a specific mixed store tends more towards a formal establishment or an informal one.

We chose to create a strict fiscal definition even though many public markets, small neighbourhood stores, specialty stores and low-budget restaurants can also be unregulated in terms of social security and tax and fiscal laws. However, despite our conservative definition, 13 % of all food and beverage purchases occur in the informal food sector. These limitations notwithstanding, we have presented the most comprehensive view of the types of food outlets from which Mexicans purchase food across different urbanicities and social strata. Strengths of the ENIGH data include the large, nationally representative sample and that purchases were assessed daily via recurring interviewer visits, that the food source was captured for each purchase and, critically, that food retailers included the formal, mixed and informal sector.

## Conclusion

In summary, small neighbourhood stores and specialty stores are the main source of food and beverage purchases in Mexico, across all income strata and levels of urbanicity. Purchases from the formal and informal sector represent the same proportion of total food purchases in Mexico. Understanding where Mexican households shop for food is relevant for designing outlet-focused food policies and interventions. Future studies should analyse how these food and beverage shopping patterns have shifted across time to quantify the ‘modernisation’ of the food environment and the scope of impact on mixed and informal outlets. Future work should also examine the quality of food and beverages across all outlets, studying how these purchases vary across urbanicity and income level.

## Supplementary material

For supplementary material accompanying this paper visit https://doi.org/10.1017/S1368980022002324. click here to view supplementary material
