# Supplementary material for: Food and beverage purchases at formal and informal outlets in Mexico
Source: Public Health Nutr. 2022 Oct 26;26(5):1034–43. doi: 10.1017/S1368980022002324 (PMC7614539; doi:10.1017/S1368980022002324)
Supplement: Supplementary file 1 [file S1368980022002324sup001.docx]

**SUPPLEMENTARY INFORMATION**

**Table S1. Mean quarterly income by urbanicity in Mexican pesos, ENIGH 2018.**

|  |  | **Rural** | **Small** | **Medium** | **Metropolitan** | **National** |
| --- | --- | --- | --- | --- | --- | --- |
| Quintile 1 |  | $8,018 | $11,882 | $14,056 | $18,408 | $12,646 |
| Quintile 2 |  | $15,138 | $21,379 | $25,065 | $31,770 | $24,116 |
| Quintile 3 |  | $22,425 | $30,623 | $36,083 | $45,458 | $35,693 |
| Quintile 4 |  | $33,022 | $43,337 | $52,076 | $66,348 | $53,134 |
| Quintile 5 |  | $71,822 | $89,498 | $106,378 | $152,534 | $122,708 |

**Figure S1. Households’ food and beverage purchases by food outlet stratified by household level size, ENIGH 2018.**


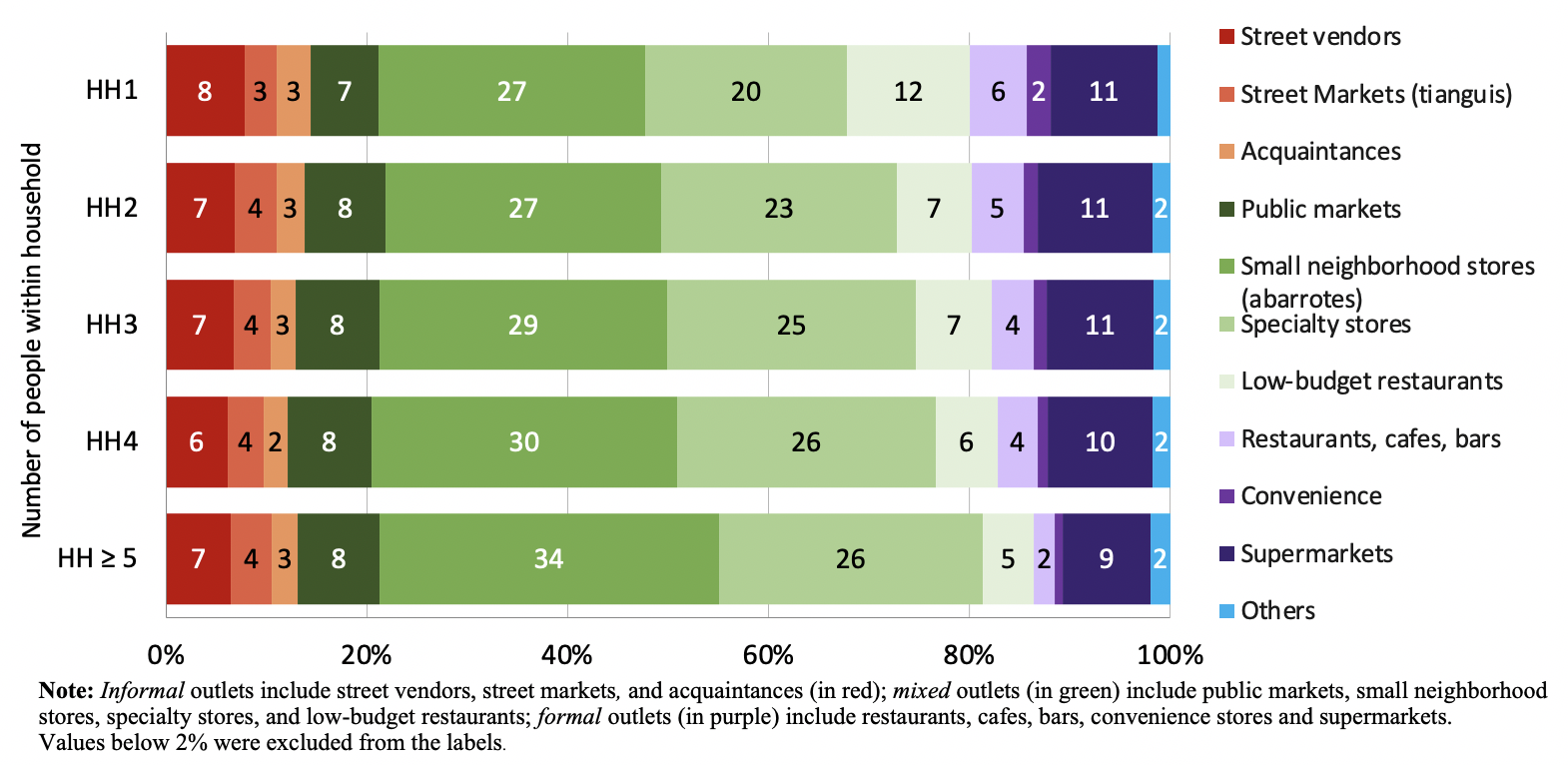


**Table S2: Standard errors of households’ food and beverage purchases by food outlet, ENIGH 2018.**

| **Food Outlet** | **Percentage** | **SE** |
| --- | --- | --- |
| Street vendors | 7 | 0.107 |
| Street Markets (*tianguis*) | 4 | 0.089 |
| Acquaintances | 3 | 0.057 |
| Public markets | 8 | 0.175 |
| Small neighborhood stores (*abarrotes*) | 30 | 0.185 |
| Specialty stores | 25 | 0.188 |
| Low-budget restaurants | 7 | 0.102 |
| Restaurants, cafes, bars | 4 | 0.092 |
| Convenience | 1 | 0.032 |
| Supermarkets | 10 | 0.124 |
| Others | 2 | 0.067 |

**Table S3: Standard errors of households’ food and beverage purchases by food outlet and urbanicity, ENIGH 2018.**

| **Food Outlet** | **Rural** | | **Small** | | **Medium** | | **Metropolitan** | |
| --- | --- | --- | --- | --- | --- | --- | --- | --- |
|  | **%** | SE | **%** | SE | **%** | SE | **%** | SE |
| Street vendors | 10 | 0.269 | 7 | 0.316 | 6 | 0.267 | 5 | 0.133 |
| Street Markets (*tianguis*) | 4 | 0.206 | 4 | 0.239 | 2 | 0.197 | 4 | 0.125 |
| Acquaintances | 5 | 0.163 | 4 | 0.209 | 2 | 0.135 | 1 | 0.045 |
| Public markets | 5 | 0.233 | 7 | 0.565 | 11 | 0.515 | 9 | 0.260 |
| Small neighborhood stores (*abarrotes*) | 43 | 0.467 | 35 | 0.616 | 28 | 0.425 | 23 | 0.214 |
| Specialty stores | 19 | 0.324 | 29 | 0.565 | 29 | 0.575 | 25 | 0.265 |
| Low-budget restaurants | 4 | 0.122 | 6 | 0.258 | 7 | 0.299 | 8 | 0.166 |
| Restaurants, cafes, bars | 1 | 0.054 | 2 | 0.135 | 3 | 0.213 | 6 | 0.172 |
| Convenience | 0 | 0.017 | 0 | 0.045 | 1 | 0.091 | 2 | 0.058 |
| Supermarkets | 4 | 0.148 | 5 | 0.271 | 10 | 0.319 | 15 | 0.214 |
| Others | 5 | 0.269 | 1 | 0.069 | 1 | 0.059 | 1 | 0.043 |

**Table S4: Standard errors of households’ food and beverage purchases by food outlet and income level, ENIGH 2018.**

| **Food Outlet** | **Q1** | | **Q2** | | **Q3** | | **Q4** | | **Q5** | |
| --- | --- | --- | --- | --- | --- | --- | --- | --- | --- | --- |
|  | **%** | SE | **%** | SE | **%** | SE | **%** | SE | **%** | SE |
| Street vendors | 9 | 0.217 | 7 | 0.180 | 7 | 0.170 | 6 | 0.183 | 5 | 0.152 |
| Street Markets (*tianguis*) | 3 | 0.181 | 4 | 0.144 | 4 | 0.144 | 4 | 0.167 | 3 | 0.147 |
| Acquaintances | 5 | 0.169 | 3 | 0.093 | 2 | 0.075 | 2 | 0.073 | 2 | 0.072 |
| Public markets | 7 | 0.280 | 8 | 0.277 | 9 | 0.307 | 9 | 0.283 | 7 | 0.264 |
| Small neighborhood stores (*abarrotes*) | 42 | 0.441 | 35 | 0.320 | 30 | 0.281 | 26 | 0.267 | 17 | 0.242 |
| Specialty stores | 21 | 0.347 | 26 | 0.313 | 27 | 0.305 | 26 | 0.324 | 24 | 0.319 |
| Low-budget restaurants | 4 | 0.146 | 6 | 0.172 | 7 | 0.207 | 8 | 0.220 | 10 | 0.252 |
| Restaurants, cafes, bars | 1 | 0.064 | 1 | 0.073 | 2 | 0.106 | 4 | 0.140 | 11 | 0.339 |
| Convenience | 1 | 0.042 | 1 | 0.058 | 1 | 0.052 | 2 | 0.066 | 2 | 0.079 |
| Supermarkets | 4 | 0.148 | 7 | 0.192 | 10 | 0.217 | 12 | 0.244 | 17 | 0.315 |
| Others | 4 | 0.243 | 2 | 0.083 | 1 | 0.051 | 1 | 0.058 | 1 | 0.085 |

**Table S5: Standard errors of households’ food and beverage purchases by food outlet, urbanicity and income, ENIGH 2018.**

| **RURAL** | | | | | | | | | | | |
| --- | --- | --- | --- | --- | --- | --- | --- | --- | --- | --- | --- |
| **Standard Errors** | Street vendors | Street markets (*tianguis*) | Acquaintances | Public markets | Small neighborhood stores (*abarrotes*) | Specialty stores | Low-budget restaurants | Restaurants, cafes, bars | Convenience | Supermarkets | Others |
| Q1 | 0.510 | 0.397 | 0.333 | 0.386 | 0.991 | 0.509 | 0.178 | 0.050 | 0.022 | 0.135 | 0.718 |
| Q2 | 0.389 | 0.323 | 0.313 | 0.341 | 0.683 | 0.448 | 0.185 | 0.063 | 0.026 | 0.138 | 0.397 |
| Q3 | 0.335 | 0.259 | 0.235 | 0.339 | 0.619 | 0.467 | 0.206 | 0.071 | 0.029 | 0.221 | 0.265 |
| Q4 | 0.331 | 0.265 | 0.182 | 0.311 | 0.554 | 0.438 | 0.262 | 0.083 | 0.030 | 0.250 | 0.205 |
| Q5 | 0.345 | 0.264 | 0.190 | 0.339 | 0.592 | 0.443 | 0.288 | 0.199 | 0.047 | 0.328 | 0.198 |
| **SMALL** | | | | | | | | | | | |
| **Standard Errors** | Street vendors | Street Markets (*tianguis*) | Acquaintances | Public markets | Small neighborhood stores (*abarrotes*) | Specialty stores | Low-budget restaurants | Restaurants, cafes, bars | Convenience | Supermarkets | Others |
| Q1 | 0.472 | 0.344 | 0.570 | 0.816 | 1.337 | 1.314 | 0.447 | 0.115 | 0.034 | 0.327 | 0.180 |
| Q2 | 0.538 | 0.425 | 0.387 | 0.767 | 1.064 | 0.930 | 0.444 | 0.142 | 0.111 | 0.327 | 0.124 |
| Q3 | 0.335 | 0.351 | 0.224 | 0.800 | 0.763 | 0.879 | 0.520 | 0.228 | 0.070 | 0.543 | 0.110 |
| Q4 | 0.453 | 0.400 | 0.220 | 0.725 | 0.879 | 0.745 | 0.607 | 0.280 | 0.145 | 0.494 | 0.109 |
| Q5 | 0.661 | 0.463 | 0.240 | 0.558 | 0.740 | 0.720 | 0.545 | 0.349 | 0.071 | 0.574 | 0.130 |
| **MEDIUM** | | | | | | | | | | | |
| **Standard Errors** | Street vendors | Street Markets (*tianguis*) | Acquaintances | Public markets | Small neighborhood stores (*abarrotes*) | Specialty stores | Low-budget restaurants | Restaurants, cafes, bars | Convenience | Supermarkets | Others |
| Q1 | 0.460 | 0.469 | 0.323 | 0.914 | 0.849 | 0.867 | 0.404 | 0.167 | 0.133 | 0.498 | 0.066 |
| Q2 | 0.445 | 0.315 | 0.218 | 0.821 | 0.786 | 0.888 | 0.540 | 0.204 | 0.185 | 0.555 | 0.079 |
| Q3 | 0.497 | 0.309 | 0.248 | 0.827 | 0.771 | 0.850 | 0.527 | 0.249 | 0.172 | 0.555 | 0.096 |
| Q4 | 0.318 | 0.338 | 0.222 | 0.767 | 0.688 | 0.997 | 0.504 | 0.346 | 0.102 | 0.616 | 0.111 |
| Q5 | 0.361 | 0.250 | 0.170 | 0.526 | 0.550 | 0.801 | 0.703 | 0.728 | 0.184 | 0.671 | 0.170 |
| **METROPOLITAN** | | | | | | | | | | | |
| **Standard Errors** | Street vendors | Street Markets (*tianguis*) | Acquaintances | Public markets | Small neighborhood stores (*abarrotes*) | Specialty stores | Low-budget restaurants | Restaurants, cafes, bars | Convenience | Supermarkets | Others |
| Q1 | 0.259 | 0.236 | 0.108 | 0.463 | 0.462 | 0.458 | 0.297 | 0.138 | 0.115 | 0.356 | 0.061 |
| Q2 | 0.251 | 0.231 | 0.097 | 0.474 | 0.411 | 0.466 | 0.312 | 0.168 | 0.090 | 0.347 | 0.068 |
| Q3 | 0.261 | 0.232 | 0.078 | 0.471 | 0.360 | 0.450 | 0.314 | 0.206 | 0.112 | 0.373 | 0.056 |
| Q4 | 0.243 | 0.261 | 0.095 | 0.414 | 0.361 | 0.438 | 0.364 | 0.340 | 0.133 | 0.429 | 0.075 |
| Q5 | 0.210 | 0.197 | 0.099 | 0.393 | 0.292 | 0.466 | 0.368 | 0.568 | 0.115 | 0.480 | 0.157 |
