# Supplementary material for: Food and beverage purchases at formal and informal outlets in Mexico
Source: Public Health Nutr. Author manuscript; Available in PMC 2023 May 15. (PMC7614539; doi:10.1017/S1368980022002324)
Supplement: Supplementary file 1 [file EMS156842-supplement-supplementary_Material.pdf]

Supplementary material

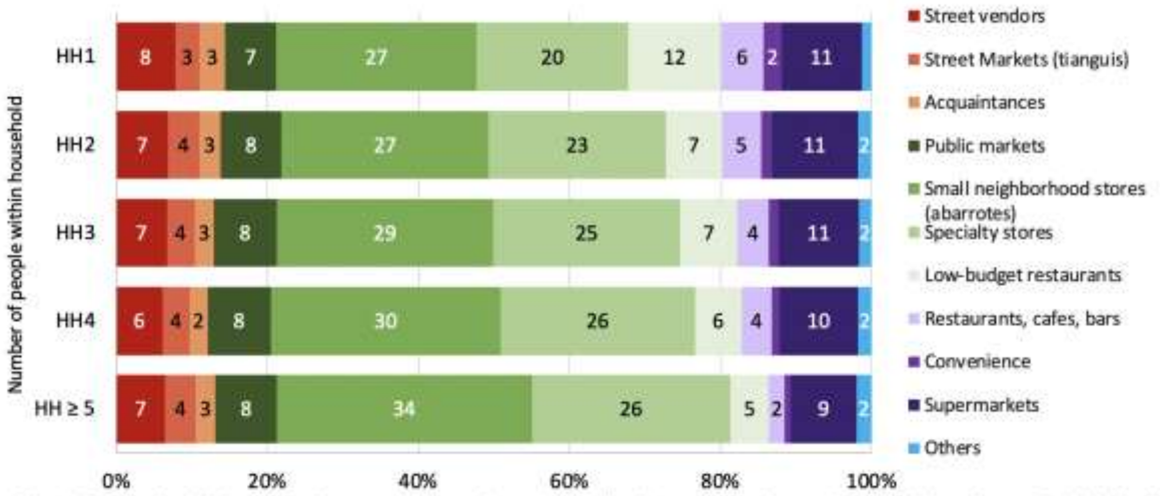

Note: *Informal* outlets include street vendors, street markets, and acquaintances (in red); *mixed* outlets (in green) include public markets, small neighborhood stores, specialty stores, and low-budget restaurants; *formal* outlets (in purple) include restaurants, cafes, bars, convenience stores and supermarkets. Values below 2% were excluded from the labels.

Figure S1. Households' food and beverage purchases by food outlet stratified by household level size, ENIGH 2018.

https://doi.org/10.1017/S1368980022002324 Published online by Cambridge University Press

Accepted manuscript

Table S1. Mean quarterly income by urbanicity in Mexican pesos, ENIGH 2018.

	**Rural**	**Small**	**Medium**	**Metropolitan**	**National**
Quintile 1	$8,018	$11,882	$14,056	$18,408	$12,646
Quintile 2	$15,138	$21,379	$25,065	$31,770	$24,116
Quintile 3	$22,425	$30,623	$36,083	$45,458	$35,693
Quintile 4	$33,022	$43,337	$52,076	$66,348	$53,134
Quintile 5	$71,822	$89,498	$106,378	$152,534	$122,708

https://doi.org/10.1017/S1368980022002324 Published online by Cambridge University Press

Table S2. Standard errors of households' food and beverage purchases by food outlet, ENIGH 2018.

**Food Outlet**	**Percentage**	**SE**
Street vendors	7	0.107
Street Markets (*tianguis*)	4	0.089
Acquaintances	3	0.057
Public markets	8	0.175
Small neighborhood stores (*abarrotes*)	30	0.185
Specialty stores	25	0.188
Low-budget restaurants	7	0.102
Restaurants, cafes, bars	4	0.092
Convenience	1	0.032
Supermarkets	10	0.124
Others	2	0.067

https://doi.org/10.1017/S1368980022002324 Published online by Cambridge University Press

Table S3. Standard errors of households' food and beverage purchases by food outlet and urbanicity, ENIGH 2018.

**Food Outlet**	**Rural**		**Small**		**Medium**		**Metropolitan**	
	**%**	SE	**%**	SE	**%**	SE	**%**	SE
Street vendors	10	0.269	7	0.316	6	0.267	5	0.133
Street Markets (*tianguis*)	4	0.206	4	0.239	2	0.197	4	0.125
Acquaintances	5	0.163	4	0.209	2	0.135	1	0.045
Public markets	5	0.233	7	0.565	11	0.515	9	0.260
Small neighborhood stores (*abarrotes*)	43	0.467	35	0.616	28	0.425	23	0.214
Specialty stores	19	0.324	29	0.565	29	0.575	25	0.265
Low-budget restaurants	4	0.122	6	0.258	7	0.299	8	0.166
Restaurants, cafes, bars	1	0.054	2	0.135	3	0.213	6	0.172
Convenience	0	0.017	0	0.045	1	0.091	2	0.058
Supermarkets	4	0.148	5	0.271	10	0.319	15	0.214
Others	5	0.269	1	0.069	1	0.059	1	0.043

https://doi.org/10.1017/S1368980022002324 Published online by Cambridge University Press

Table S4. Standard errors of households' food and beverage purchases by food outlet and income level, ENIGH 2018.

**Food Outlet**	**Q1**		**Q2**		**Q3**		**Q4**		**Q5**	
	**%**	SE	**%**	SE	**%**	SE	**%**	SE	**%**	SE
Street vendors	9	0.217	7	0.180	7	0.170	6	0.183	5	0.152
Street Markets (*tianguis*)	3	0.181	4	0.144	4	0.144	4	0.167	3	0.147
Acquaintances	5	0.169	3	0.093	2	0.075	2	0.073	2	0.072
Public markets	7	0.280	8	0.277	9	0.307	9	0.283	7	0.264
Small neighborhood stores (*abarrotes*)	42	0.441	35	0.320	30	0.281	26	0.267	17	0.242
Specialty stores	21	0.347	26	0.313	27	0.305	26	0.324	24	0.319
Low-budget restaurants	4	0.146	6	0.172	7	0.207	8	0.220	10	0.252
Restaurants, cafes, bars	1	0.064	1	0.073	2	0.106	4	0.140	11	0.339
Convenience	1	0.042	1	0.058	1	0.052	2	0.066	2	0.079
Supermarkets	4	0.148	7	0.192	10	0.217	12	0.244	17	0.315
Others	4	0.243	2	0.083	1	0.051	1	0.058	1	0.085

https://doi.org/10.1017/S1368980022002324 Published online by Cambridge University Press

Table S5. Standard errors of households' food and beverage purchases by food outlet, urbanicity and income, ENIGH 2018.

RURAL											
**Standard Errors**	Street vendors	Street markets (*tianguis*)	Acquaintances	Public markets	Small neighborhood stores (*abarrotes*)	Specialty stores	Low-budget restaurants	Restaurants, cafes, bars	Convenience	Supermarkets	Others
Q1	0.510	0.397	0.333	0.386	0.991	0.509	0.178	0.050	0.022	0.135	0.718
Q2	0.389	0.323	0.313	0.341	0.683	0.448	0.185	0.063	0.026	0.138	0.397
Q3	0.335	0.259	0.235	0.339	0.619	0.467	0.206	0.071	0.029	0.221	0.265
Q4	0.331	0.265	0.182	0.311	0.554	0.438	0.262	0.083	0.030	0.250	0.205
Q5	0.345	0.264	0.190	0.339	0.592	0.443	0.288	0.199	0.047	0.328	0.198
**SMALL**											
**Standard Errors**	Street vendors	Street Markets (*tianguis*)	Acquaintances	Public markets	Small neighborhood stores (*abarrotes*)	Specialty stores	Low-budget restaurants	Restaurants, cafes, bars	Convenience	Supermarkets	Others
Q1	0.472	0.344	0.570	0.816	1.337	1.314	0.447	0.115	0.034	0.327	0.180
Q2	0.538	0.425	0.387	0.767	1.064	0.930	0.444	0.142	0.111	0.327	0.124
Q3	0.335	0.351	0.224	0.800	0.763	0.879	0.520	0.228	0.070	0.543	0.110
Q4	0.453	0.400	0.220	0.725	0.879	0.745	0.607	0.280	0.145	0.494	0.109
Q5	0.661	0.463	0.240	0.558	0.740	0.720	0.545	0.349	0.071	0.574	0.130
**MEDIUM**											
**Standard Errors**	Street vendors	Street Markets (*tianguis*)	Acquaintances	Public markets	Small neighborhood stores (*abarrotes*)	Specialty stores	Low-budget restaurants	Restaurants, cafes, bars	Convenience	Supermarkets	Others
Q1	0.460	0.469	0.323	0.914	0.849	0.867	0.404	0.167	0.133	0.498	0.066
Q2	0.445	0.315	0.218	0.821	0.786	0.888	0.540	0.204	0.185	0.555	0.079
Q3	0.497	0.309	0.248	0.827	0.771	0.850	0.527	0.249	0.172	0.555	0.096
Q4	0.318	0.338	0.222	0.767	0.688	0.997	0.504	0.346	0.102	0.616	0.111
Q5	0.361	0.250	0.170	0.526	0.550	0.801	0.703	0.728	0.184	0.671	0.170
**METROPOLITAN**											
**Standard Errors**	Street vendors	Street Markets (*tianguis*)	Acquaintances	Public markets	Small neighborhood stores (*abarrotes*)	Specialty stores	Low-budget restaurants	Restaurants, cafes, bars	Convenience	Supermarkets	Others
Q1	0.259	0.236	0.108	0.463	0.462	0.458	0.297	0.138	0.115	0.356	0.061
Q2	0.251	0.231	0.097	0.474	0.411	0.466	0.312	0.168	0.090	0.347	0.068
Q3	0.261	0.232	0.078	0.471	0.360	0.450	0.314	0.206	0.112	0.373	0.056
Q4	0.243	0.261	0.095	0.414	0.361	0.438	0.364	0.340	0.133	0.429	0.075
Q5	0.210	0.197	0.099	0.393	0.292	0.466	0.368	0.568	0.115	0.480	0.157

https://doi.org/10.1017/S1368980022002324 Published online by Cambridge University Press
